# Can Different Impression Techniques Affect the Chewing Efficiency of Mandibular Implant-Retained Overdentures?

**DOI:** 10.1590/0103-6440202405941

**Published:** 2024-10-28

**Authors:** Esmail Ahmed Abdel-Gawwad, Mostafa I. Fayad, Mohamed Abdullah Quassem, Mohamed Osman, Wesam E. Badr, Hamada Z Mahross

**Affiliations:** 1 Department of Removable Prosthodontics, Faculty of Dental Medicine, Al-Azhar University, Cairo, Egypt; 2 Department of Substitutive Dental Science, College of Dentistry, Taibah University, Saudi Arabia. Department of Removable Prosthodontics, Faculty of Dental Medicine, Al-Azhar University, Cairo, Egypt; 3 Department of Removable Prosthodontics, Faculty of Dental Medicine, Al-Azhar University, Cairo, Egypt. Department of Substitutive Dental Sciences, College of Dentistry & Pharmacy, Buraydah Private Colleges, Buraydah, Saudi Arabia

**Keywords:** Chewing Efficiency, Implant-Retained Overdentures, Impression Techniques

## Abstract

Several impression techniques and theories have been developed for implant-retained overdentures, each with its own set of advantages and limitations. This study aimed to assess the chewing efficiency of mandibular implant-retained overdentures fabricated using three distinct impression techniques (mucofunctional, selective, and minimal pressure impression techniques). Twenty-six patients with complete edentulism free from any oral or systemic disease were selected to participate in the study. Three complete mandibular implant-retained overdentures were constructed for each patient, and grouped into three groups according to the impression technique employed in overdenture construction; Group A: Patients were treated by mandibular implant-retained overdenture fabricated using mucofunctional impression technique; Group B: Patients were treated by mandibular implant-retained overdenture fabricated using selective pressure impression technique; Group C: Patients were treated by mandibular implant-retained overdenture fabricated using minimal pressure impression technique. Chewing efficiency was evaluated for each patient with the implant-retained mandibular overdenture after three months of prosthesis insertion as an adaptation period. The data were collected, tabulated, and statistically analyzed. Results indicated that mandibular implant-retained overdentures made using the mucofunctional impression technique showed higher mean values of chewing efficiency parameters than mandibular overdentures made using selective and minimal pressure impression techniques. Within the limitations of this study, it can be concluded that the mucofunctional impression technique may be effective in improving the chewing efficiency of mandibular implant-retained overdentures more than minimal or selective pressure impression techniques.

## Resumo

Diversas técnicas e teorias de moldagem foram desenvolvidas para overdentaduras retidas por implantes, cada uma com seu próprio conjunto de vantagens e limitações. Este estudo teve como objetivo avaliar a eficiência mastigatória de overdentaduras mandibulares retidas por implantes fabricados utilizando três técnicas distintas de moldagem (moldagem mucosa funcional, seletiva e de pressão mínima). Vinte e seis pacientes com edentulismo total, livres de qualquer doença oral ou sistêmica, foram selecionados para participar do estudo. Três overdentaduras mandibulares completas retidas por implantes foram construídas para cada paciente e agrupadas em três grupos de acordo com a técnica de moldagem empregada na construção das overdentaduras; Grupo A: Pacientes tratados com overdentaduras mandibulares retidas por implantes fabricadas utilizando a técnica de moldagem mucosa funcional; Grupo B: Pacientes tratados com overdentaduras mandibulares retidas por implantes fabricadas utilizando a técnica de moldagem de pressão seletiva; Grupo C: Pacientes tratados com overdentaduras mandibulares retidas por implantes fabricadas utilizando a técnica de moldagem de pressão mínima. A eficiência mastigatória foi avaliada para cada paciente com a overdentadura mandibular retida por implantes após três meses de inserção da prótese como período de adaptação. Os dados foram coletados, tabulados e analisados estatisticamente. Os resultados indicaram que as overdentaduras mandibulares retidas por implantes feitas utilizando a técnica de moldagem mucosa funcional apresentaram valores médios mais altos nos parâmetros de eficiência mastigatória do que as overdentaduras mandibulares feitas utilizando as técnicas de moldagem de pressão seletiva e mínima. Dentro das limitações deste estudo, pode-se concluir que a técnica de moldagem mucosa funcional pode ser eficaz na melhoria da eficiência mastigatória de overdentaduras mandibulares retidas por implantes mais do que as técnicas de moldagem de pressão mínima ou seletiva.

## Introduction

The prosthetic management of edentulous patients has been a significant challenge in dental practice for a long period. The standard care for these patients has been complete maxillary and mandibular dentures. However, many patients encounter difficulties in adapting to their mandibular dentures, which can be attributed to issues such as lack of comfort, retention, stability, and chewing function. Notably, the lack of retention and stability is one of the major complaints among edentulous individuals ^1^ ^,^ ^2^.

In recent years, implant-retained overdentures have emerged as a promising alternative to conventional complete dentures. This treatment modality offers several advantages, including increased comfort, improved chewing efficiency, greater patient satisfaction, preservation of the residual ridge, enhanced retention and stability, and an overall improvement in quality of life ^3^. The success of implant-retained overdentures relies on various factors, including implant positioning, prosthesis design, and impression technique. Among these factors, the impression technique plays a critical role in ensuring accurate and precise fitting of the overdenture, which directly impacts masticatory efficiency and patient comfort ^4^.

Several studies have highlighted the benefits of implant-retained mandibular overdentures compared to conventional dentures where the provision of two mandibular implants significantly enhances chewing function and patients’ quality of life. Additionally, implant-retained overdentures offer objective benefits in masticatory performance, particularly for patients with marked mandibular resorption and difficulty adapting to mandibular dentures. They provide improved stability, retention, and dramatic improvements in mastication and speech. Moreover, implant-retained mandibular overdentures allow for better chewing function compared to conventional dentures ^5^ ^,^ ^6^ ^,^ ^7^.

Mastication, the process of breaking down food mechanically, is considered the initial step in the digestive process and it is regulated by the central pattern generator located in the brainstem. The efficiency of mastication is influenced by the way food is chewed, as this affects the subsequent enzymatic processing in the digestive system. The chewing function can be assessed by the individual's ability to grind natural or artificial test foods ^8^ ^,^ ^9^ ^,^ ^10^. Improving chewing function with implant retained overdenture is a key goal in the rehabilitation of completely edentulous patients. The ability to chew and process food effectively not only enhances the nutritional intake but also contributes to overall oral health and well-being ^11^ ^,^ ^12^.

Several impression techniques have been developed for implant-retained overdentures, each with its own set of indications and limitations. The selection of an impression technique is dictated by factors such as the number and distribution of implants, the presence of anatomical undercuts, and the clinician's preference. Many authors have discussed the controversy regarding the pressure exerted while making the impression of complete dentures ^13^.

The commonly used impression techniques are mucofunctional, selective pressure, and minimal pressure impression techniques. The mucofunctional impression technique involves recording the functional movements of the oral tissues during impression-making, aiming to reproduce the dynamic aspects of the oral cavity. The selective pressure impression technique focuses on applying pressure selectively to specific areas to achieve accurate tissue support. The minimal pressure impression technique aims to minimize pressure on the soft tissues during impression making ^14^ ^,^ ^15^ ^,^ ^16^. 

Although the previous techniques can be used for taking the impressions of implant overdentures, limited research has been conducted to compare the masticatory efficiency of overdentures constructed using these different impression techniques. Therefore, the study aimed to evaluate the impact of different impression techniques on the chewing efficiency of implant-retained mandibular overdenture. The working hypothesis of the study was that the type of impression technique does not influence the chewing efficiency.

## Material and Methods

Twenty-six participants with complete edentulism (17 males and 9 females), aged 47 to 69 years, were randomly selected among the patients referred to the outpatient clinic of Removable Prosthodontic Department, Faculty of Dental Medicine, Al-Azhar University, Cairo, Egypt. Based on a prior study by Bhat et al. ^15^ using SPSS software version 20, the sample's statistical power was determined to be 80%. Subsequently, a sample comprising ^26^ ^)^ individuals was selected for the study. The research proposal received approval from the Research Ethics Committee of Al-Azhar University, Faculty of Dental Medicine, under reference number (448-10-12). The patients were enrolled in the study after signing a consent form and explaining all surgical and other interventional procedures.

The selection criteria for patients enrolled in this study were as follows: complete edentulism for a minimum of six months before implant placement to allow for complete bone remodeling ^17^, normal maxillomandibular relations, absence of temporomandibular disorders and neuromuscular disorders, no systemic conditions that could influence the prognosis of implant-overdenture, healthy mucosa covering the edentulous ridge without any remaining roots, cysts, residual infection, or impacted teeth, a cooperative patient who could maintain good oral hygiene, sufficient bone volume at the intended implant site, non-smoker, non-use of narcotic drugs and alcohol, absence of abnormal detrimental habits such as bruxism and clenching, and no prior use of dentures.

### Grouping

For each patient, three complete mandibular implant-retained overdentures were constructed and grouped into three groups according to the impression technique used.

Group A: The patients received mandibular implant-retained overdentures constructed using the mucofunctional impression technique.

Group B: The patients received mandibular implant-retained overdentures constructed using the selective pressure impression technique.

Group C: The patients received mandibular implant-retained overdentures constructed using the minimal pressure impression technique.

A cone beam computed tomography (CBCT) scan was performed to evaluate bone density and quality prior to implant placement. An irreversible hydrocolloid impression was made using Alginate impression material; CA37 (Cavex Holland BV Co., CJ Haarlem, The Netherlands), and poured into dental stone to create a study cast. An acrylic transparent surgical guide template was then constructed to allow optimal implant placement with proper angulation.

### Surgical implant placement

Surgery began with the use of a specific surgical kit (Slimline, Dentium surgical kit) and a surgical guide under mandibular nerve block anesthesia using ARTINIBSA 40 mg/ml anesthetic solution for injection (Inibsa Dental Co., Barcelona, Spain). The procedure began by creating an initial opening with a 1.1 mm diameter pilot drill, which was the only end-cutting bur used. It was crucial to establish proper positioning during the use of the pilot drill.

Subsequent surgical drills were used to widen the osteotomy site, and the surgical procedures were completed by employing the specific drills guided using the surgical guide. The chosen implant system exhibited superior quality, remarkable initial stability, and simple prosthetic applications; specifically, the Slimline implant system (Dentium Co., Ltd., Seoul, South Korea), which features a one-piece dental implant with a ball-type abutment. The implants were accurately placed in the bilateral canine region using a one-piece type, and the ball and socket attachments provided excellent retention. The procedure was completed using a 1-stage surgical technique, and loading was planned after three months of implant placement (delayed loading protocol). Participants were instructed to adhere to a soft diet ^18^.

### Final overdenture impressions

A preliminary impression of the mandibular arch was obtained using Alginate irreversible hydrocolloid material; CA37, Cavex, and poured into dental stone to obtain a study cast on which three custom impression trays were made using Trayplast cold-cure acrylic resin (Vertex-Dental BV Co., Zeist, Netherland). The trays were designed with an open area in the implant region. Impression transfer copings were secured to the implants. For each patient, three different impressions were made using the three different final impression techniques at 48-hour intervals to facilitate recovery of the mucosal tissue ^19^.

A. Mucofunctional impression technique ^20^: The custom impression tray was constructed without spacers or stoppers, with an occlusion rim made at the correct vertical dimension and then border molded using a low-fusing compound (Impression Compound; Kerr Dental Italia S.pA, Salerno, Italy). Following the border molding process, the final impression wash was obtained using zinc oxide eugenol impression paste (S.S. White Manufacturing LTD Co., Gloucester, England) at the appropriate vertical dimension using a closed-mouth technique. After the tray was removed, the excess impression material was trimmed from the open areas of the tray using a scalpel. Subsequently, the tray was repositioned over the alveolar ridge. The light-body polyvinyl siloxane material, Oranwash L (Zhermack, Badia Polesine, Italy), was applied around the impression copings while firm pressure was exerted on the distal area of the tray.

B. Selective pressure impression technique ^21^: During the fabrication of the custom tray, it was relieved by applying a thin layer of baseplate wax, excluding the buccal shelves and retromolar pad areas. Subsequently, border molding of the tray was performed using a low-fusing compound, which aims to capture the peripheral extension of the tray and the shaping of the borders to replicate the functional contours of the oral tissues. The final impression wash was then completed using medium-consistency condensation silicone impression material (Thixoflex M, Zhermack, ITALY).

C. Minimal pressure impression technique ^22^: The custom impression tray was constructed and relieved with a 2-mm wax spacer and incorporated two stoppers positioned bilaterally at the premolar and molar areas. Perforations were made in the custom tray to facilitate the escape of excess impression material, and it was border molded using a low-fusing compound. The final impression wash was obtained using light-body polyvinyl siloxane material (Oranwash L, Zhermack, ITALY) while applying minimal pressure to ensure anatomic recording of the denture-bearing area.

For all three impression techniques, after setting, the impression was carefully removed and checked. The implant analog was securely fastened and positioned in its place within the impression and then poured to obtain the master cast. Maxillomandibular relationships were recorded using the inter-occlusal wax method, and the casts were mounted on a semi-adjustable articulator; A7 Plus (Bio-Art Co., São Carlos, SP, Brazil). Acrylic resin artificial teeth with a 20-degree cusp angle (Yamahachi Dental MFG Co., Nishiuracho Gamagori, Japan) were arranged based on the bilateral balanced occlusal concept. Subsequently, waxing-up procedures were performed. The waxed-up denture was then evaluated intraorally, and any mandatory modifications were made to ensure proper fit and function.

### Denture duplication

The process of replicating the polished and occlusal surfaces of the mandibular overdenture involved specific steps using a traditional flask. A heavy consistency polysiloxane impression material (Zetaplus Putty, Zhermack, ITALY) was used to create a mold of the polished surface by applying it to the counter-die part of the flask and positioning the waxed-up denture against it. The cast obtained from an alternative impression technique was placed in the die part of the flask. Acrylic resin artificial teeth of similar size were inserted into the mold and correctly aligned. Molten baseplate wax was carefully poured into the void between the teeth and the cast to replicate the original denture's contour and volume. The fabrication of the three waxed-up mandibular overdentures was done simultaneously by a skilled technician, following the manufacturer's guidelines for using and processing acrylic resin. After completing the denture fabrication, it was delivered to the patient following clinical remounting procedures. The patient was provided with instructions on how to properly insert and remove the prosthesis and was educated about the importance of maintaining good oral and denture hygiene. A three-month adaptation period was provided before assessing the chewing efficiency of each overdenture, allowing for neuromuscular adaptation and control of the new prosthesis.

### Chewing efficiency assessment 

Chewing efficiency was assessed following the methodology outlined by Feine et al and Khamis et al ^23^ ^,^ ^24^. A one cubic centimeter portion of raw carrot, peanut, and apple was chewed and swallowed at a normal pace, and the following parameters were recorded: the number of chewing strokes until the first swallow, the number of chewing strokes until the mouth was clear of food, the number of swallows until the mouth was clear of food, the duration in seconds until the first swallow, and the duration in seconds until the mouth was clear of food. This process was repeated with five cubes of each food, and the mean of the five measurements was utilized for analysis. The chewing efficiency of each patient with the implant-retained overdenture was assessed three months after overdenture insertion, serving as an adaptation period.

### Statistical analysis

The data were collected, tabulated, and analyzed statistically using SPSS© Statistics for Windows version 20.0 software (IBM Corp., NY, USA). The data were expressed as mean and standard deviation (SD) values. Repeated measures ANOVA test was used to compare the mean values of different chewing parameters for the three impression techniques using different food types. Post-hoc Tukey’s test was employed for pairwise comparisons between the mean values when the ANOVA test yielded statistical significance, and the significance level was set at P ≤ 0.05.

## Results

The mean values for different parameters of chewing efficiency, comparing the different impression techniques employed in overdenture construction using three types of test food (apple, peanut, and carrot), are displayed in Table 1. These parameters include the following: the number of chewing cycles until the first swallow, the number of chewing cycles until the mouth was clear of food, the number of swallows until the mouth was clear of food, the time in seconds passed until the first swallow, and the time in seconds passed until mouth was clear of food.

The results showed that the mucofunctional impression group demonstrated higher mean values for the different parameters of chewing efficiency compared to the selective and minimal pressure impression groups. There was also a significant difference between mean values of chewing parameters among the three groups of impression techniques (p-value <0.05) except for mean values of the number of swallows till empty mouth where p>0.05.

**Table 1:** Comparison of mean values and (SD; standard deviation) of different chewing efficiency parameters among the three impression groups.

*P-value*	Minimal pressure technique	Selective pressure technique	Mucofunctional impression technique	*Test food type*	Impression techniques/ Chewing efficiency parameters
<0.001*	15.5 ^a^ (1.1)	14.5 ^a^ (1.7)	12.9 ^b^ (1.5)	*Apple*	Number of strokes to the first swallow
0.002*	18.0 ^a^ (1.8)	17.9 ^a^ (2.6)	17.2 ^b^ (2.2)	*Peanut*	
0.001*	32.6 ^a^ (2.7)	31.8 ^b^ (3.2)	31.1 ^c^ (2.9)	*Carrot*	
<0.001*	21.0 ^a^ (2.5)	19.4 ^b^ (1.5)	18.3 ^b^ (2.8)	*Apple*	Number of strokes to mouth empty
0.001*	32.3 ^a^ (2.4)	31.7 ^b^ (2.8)	30.5 ^b^ (3.5)	*Peanut*	
<0.001*	52.0 ^a^ (3.5)	51.1 ^b^ (2.3)	50.0 ^c^ (3.2)	*Carrot*	
*0.069*	2.7 (0.6)	2.5 (0.9)	2.3 (1.4)	*Apple*	Number of swallows to mouth empty
*0.57*	3.5 (1.3)	2.9 (1.2)	2.8 (0.9)	*Peanut*	
*0.71*	4.2 (1.2)	3.8 (1.5)	3.2 (0.7)	*Carrot*	
0.001*	5.7 ^a^ (1.5)	5.4 ^b^ (1.8)	5.1 ^c^ (1.7)	*Apple*	Time to first swallow (sec.)
<0.001*	15.6 ^a^ (2.2)	14.4 ^b^ (1.3)	13.4 ^c^ (2.4)	*Peanut*	
<0.001*	22.9 ^a^ (1.6)	21.8 ^b^ (1.9)	21.2 ^c^ (2.3)	*Carrot*	
<0.001*	13.3 ^a^ (2.2)	12.6 ^b^ (1.3)	11.9 ^c^ (2.1)	*Apple*	Time to empty the mouth (sec.)
0.028*	20.6 ^a^ (2.5)	19.8 ^b^ (2.7)	19.6 ^b^ (1.8)	*Peanut*	
<0.001*	42.4 ^a^ (2.7)	42.0 ^a^ (4.1)	40.8 ^b^(3.3)	*Carrot*	

*: Significant at P ≤ 0.05, Different superscripts in the same row are statistically significantly different.

## Discussion

Based on an online search of scientific databases, no previous studies have compared the chewing efficiency of overdentures fabricated using different impression techniques. This is the first study to assess the difference in chewing efficiency between the minimal pressure, selective pressure, and mucofunctional impression techniques for mandibular implant-retained overdenture construction. The objective of this study was to assess the impact of different impression techniques on the chewing efficiency of implants-retained mandibular overdenture.

In the current study, two implants were utilized for retaining the overdentures. The use of implant-retained overdentures with two implants positioned in the canine region proved to be an effective approach for enhancing masticatory function in completely edentulous patients ^25^. Treatment with implant overdentures increased chewing efficiency, as evidenced by the patients' enhanced ability to chew, reduced number of chewing strokes required to reach the first swallow, and an enhanced capacity to eat tough foods after overdenture treatment. These findings were consistent with previous studies ^2^ ^,^ ^27^ ^,^ ^28^.

In the present study, three duplicate overdentures were made for each patient using three different mucosal pressure-based impression techniques. An adaptation period of three months was given before evaluation of each overdenture's chewing efficiency to allow for neuromuscular adaptation and control of the overdenture ^29^. Duplicate overdentures were made to reduce the effects of potentially confounding variables that may influence chewing efficiency among study participants like age, gender, oral health status, and systemic health of the participants, allowing comparing chewing function within participants while only changing the prosthetic techniques variable (impression techniques).

The chewing efficiency test was evaluated utilizing the methodology outlined by Feine et al ^23^ and Khamis et al ^24^, using one cubic centimeter of raw carrot, peanut, and apple as the test food. This simple and effective research approach facilitates the quantification and standardization of the chewing efficiency assessment based on objective parameters.

In the current study, there was a statistical difference between the different parameters' mean values used to measure the chewing ability of the study subjects treated with implant-retained overdentures constructed using three different impression techniques, so the null hypothesis of the study was rejected.

In the present study, the mucofunctional impression technique for implant-retained mandibular overdentures was correlated with enhanced chewing efficiency. This was evidenced by a reduced number of chewing cycles before the first swallow, a decreased number of chewing cycles required to clear the mouth of food, a minimal number of swallows necessary to clear the mouth of food, and a shorter duration until the first swallow. These findings indicate an improvement in patients' chewing efficiency and suggest the potential for a more balanced diet when compared to the minimal pressure and selective pressure impression techniques.

The mucofunctional impression technique, which focuses on capturing the functional form of the oral tissues, has shown promising results in optimizing chewing efficiency. By considering the dynamic aspects of the oral cavity during impression-making, this technique may provide a more accurate representation of the functional occlusal relationships.

A previous study by Elsyad et al. ^30^ revealed that a mucofunctional impression technique for an implant-retained mandibular overdenture is associated with a minimum deformation of the denture base during function, in contrast to the minimal pressure and selective pressure techniques which may be in harmony with the current study. This agreement may be explained by knowing that the mucofunctional technique may facilitate better occlusal contacts, and can help prevent localized stress concentration, thus improving the distribution of forces during chewing, leading to more efficient and effective mastication.

The variation in chewing efficiency among the impression techniques can be attributed to their ability to capture the precise contours of the edentulous ridge and surrounding tissues. Techniques that provide a more accurate fit tend to result in improved masticatory efficiency. Moreover, the retention and stability of the overdenture are critical factors in determining masticatory function. Different impression techniques can impact the level of retention and stability achieved. For example, a technique that enables better force distribution and optimal implant support has the potential to enhance masticatory efficiency. However, it is important to note that different impression techniques may require additional time and expertise to perform accurately. Additionally, the outcomes can be influenced by the clinician's level of experience and skill in using a specific technique.

The limitations of the study were that denture base retention and denture instability during the function weren't evaluated, as well as the number of occlusal and denture base adjustments that may vary for each overdenture which can impact chewing function outcome measures.

While this study provides valuable insights into the chewing efficiency of mandibular implant-retained overdentures, there are several areas that warrant further research: 1. Conduct studies with longer follow-up periods to assess the stability and long-term effects of different impression techniques on chewing efficiency. This will provide a more comprehensive understanding of the durability and performance of implant-retained overdentures over time. 2. Investigate the impact of different impression techniques on patient-reported outcomes such as satisfaction and quality of life. Assessing these factors can further inform treatment decisions and enhance the overall patient experience. 3. Develop evidence-based clinical guidelines that outline the most appropriate impression technique for different patient populations and clinical scenarios. These guidelines can serve as a valuable resource for clinicians and prosthodontists in decision-making and treatment planning.

## Conclusions

Within the limitations of this study, we can conclude that the chewing efficiency of implant-retained mandibular overdentures can be affected by the type of impression techniques. Comparing different techniques, the use of a mucofunctional impression technique resulted in better chewing efficiency and a slightly smaller number of chewing cycles compared to minimal or selective pressure techniques. Therefore, selecting the appropriate impression technique is crucial for achieving successful implant-retained mandibular overdentures.
